# *mulea*: An R package for enrichment analysis using multiple ontologies and empirical false discovery rate

**DOI:** 10.1186/s12859-024-05948-7

**Published:** 2024-10-18

**Authors:** Cezary Turek, Márton Ölbei, Tamás Stirling, Gergely Fekete, Ervin Tasnádi, Leila Gul, Balázs Bohár, Balázs Papp, Wiktor Jurkowski, Eszter Ari

**Affiliations:** 1 Earlham Institute, Norwich Research Park, Norwich, NR4 7UZ UK; 2 Department of Metabolism, Digestion and Reproduction, Imperial College London, The Commonwealth Building, The Hammersmith Hospital, Du Cane Road, London, W12 0NN UK; 3 Synthetic and Systems Biology Unit, Institute of Biochemistry, HUN-REN Biological Research Centre, Temesvári Krt. 62, 6726 Szeged, Hungary; 4 HCEMM-BRC Metabolic Systems Biology Research Group, Temesvári Krt. 62, 6726 Szeged, Hungary; 5 Doctoral School of Biology, University of Szeged, Közép Fasor 52, 6726 Szeged, Hungary; 6 Doctoral School of Computer Science, University of Szeged, Árpád Tér 2, 6720 Szeged, Hungary; 7 Department of Genetics, ELTE Eötvös Loránd University, Pázmány P. Stny. 1/C, 1117 Budapest, Hungary

**Keywords:** Gene set enrichment, R package, False discovery rate, Overrepresentation analysis, GMT files, Ontologies

## Abstract

Traditional gene set enrichment analyses are typically limited to a few ontologies and do not account for the interdependence of gene sets or terms, resulting in overcorrected *p*-values. To address these challenges, we introduce *mulea*, an R package offering comprehensive overrepresentation and functional enrichment analysis. *mulea* employs a progressive *empirical false discovery rate (eFDR) method*, specifically designed for interconnected biological data, to accurately identify significant terms within diverse ontologies. *mulea* expands beyond traditional tools by incorporating a wide range of ontologies, encompassing Gene Ontology, pathways, regulatory elements, genomic locations, and protein domains. This flexibility enables researchers to tailor enrichment analysis to their specific questions, such as identifying enriched transcriptional regulators in gene expression data or overrepresented protein domains in protein sets. To facilitate seamless analysis, *mulea* provides gene sets (in standardised GMT format) for 27 model organisms, covering 22 ontology types from 16 databases and various identifiers resulting in almost 900 files. Additionally, the *muleaData* ExperimentData Bioconductor package simplifies access to these pre-defined ontologies. Finally, *mulea*'s architecture allows for easy integration of user-defined ontologies, or GMT files from external sources (*e.g.,* MSigDB or Enrichr), expanding its applicability across diverse research areas. *mulea* is distributed as a CRAN *R* package downloadable from https://cran.r-project.org/web/packages/mulea/ and https://github.com/ELTEbioinformatics/mulea. It offers researchers a powerful and flexible toolkit for functional enrichment analysis, addressing limitations of traditional tools with its progressive eFDR and by supporting a variety of ontologies. Overall, *mulea* fosters the exploration of diverse biological questions across various model organisms.

**Supplementary Information:**

The online version contains supplementary material available at 10.1186/s12859-024-05948-7.

## Background

Large-scale ‘omics studies, such as transcriptomic and proteomic analyses, often generate extensive lists of genes, transcripts, or proteins exhibiting differential expression or specific characteristics. However, understanding the biological mechanisms underlying these gene lists can be challenging, and the analysis of individual genes can be insufficient for understanding complex biological processes. *Overrepresentation analysis* (ORA) and *gene set enrichment analysis* (GSEA) help extract meaningful and unbiased insights from ‘omics results by identifying shared characteristics among these lists of genes, transcripts, or proteins.

Early enrichment tools [1, 2] typically focussed on Gene Ontology (GO) [3] and/or KEGG pathway [4] enrichment, providing a way for researchers to identify overrepresented functions from curated ontologies, and help researchers move from the analysis of single genes towards more biologically relevant insights. The development of GSEA [5] constituted a substantial departure from previous approaches, as instead of relying on a threshold to designate the significance of a set, the novel method took the distribution of all genes in a ranked list into account when determining enrichment. Over time, novel resources appeared [6–8], incorporating additional gene- or protein-level properties and downstream analyses [9] to offer a deeper understanding of the studied biological systems. However, many tools still only focus on a narrow set of available ontologies or pathways, that are often predetermined by the developer, offering limited customisation options to the user, and when a larger number of ontologies are available, they are usually only so for a handful of organisms (Table 1). Additionally, most enrichment tools rely on *p*-value adjustment methods that may be too conservative for interdependent biological ontologies such as GO and, therefore may exclude important biological insights. Prompted by the lack of such comprehensive approaches, we developed the R package *mulea*: “multi-enrichment analysis”, which enables enrichment analyses using a diverse range of gene sets and ontologies for 27 model organisms, and improves the relevancy of the results by utilising a progressive *empirical false discovery rate* approach more appropriate for interdependent biological data.

*mulea* supports overrepresentation testing for a wide range of ontologies, including pathways, protein domains, genomic locations, GO terms, and gene expression regulators (such as transcription factors and microRNAs). We provide these ontologies from 16 publicly available databases, in a standardised GMT (Gene Matrix Transposed) format and through the *muleaData* ExperimentData Bioconductor package [10] for 27 model organisms, from Bacteria to human. Furthermore, *mulea* accepts the standardised GMT format, allowing easy integration of other data sources, or even user-defined ontologies.

Traditional enrichment analysis methods suffer from the overcorrection of *p*-values. Specifically, conventional *p*-value adjustment methods, like the Bonferroni [11] and Benjamini-Hochberg [12], often fail to account for the inherent interconnectedness among gene sets and ontology entries, potentially missing biologically relevant terms with significant enrichment. To address this problem, we implemented the so-called “plug-in” estimate of the false discovery rate (Algorithm 18.3 of Hastie et al*.* [13]) within the *mulea* package. This method is an *empirical*, resampling-based approach to calculating the false discovery rate (FDR), which we abbreviate as eFDR.

## Implementation

### Approaches implemented in the *mulea* package

The *mulea* R package provides two different approaches for functional enrichment analysis. For unranked sets of elements, such as significantly up- or downregulated genes, *mulea* employs the set-based overrepresentation analysis (ORA). Alternatively, if the data consists of ranked elements, for instance, genes ordered by *p*-value or log fold-change calculated by the differential expression analysis, *mulea* offers the gene set enrichment (GSEA) approach.

#### The unordered set-based overrepresentation analysis (ORA)

*Mulea* implements a set-based enrichment analysis approach that utilises the hypergeometric test (which is analogous to the one-tailed Fisher’s exact test) to identify statistically significant overrepresentation of elements from a target set (*e.g.*, significantly up- or downregulated genes) within a background set (*e.g.*, all genes that were investigated in the experiment). Therefore, a predefined threshold value—such as 0.05 for the corrected *p*-values and *z*-scores, or twofold change—has to be used in the preceding analysis.

#### Addressing multiple testing: *p*-value correction and eFDR in the ORA analysis

Performing numerous statistical tests, such as evaluating enrichment across all ontology entries, leads to an inflated number of significant results (*p*-values < 0.05) due to chance, even if all null hypotheses are true. This phenomenon, known as the multiple testing problem, necessitates *p*-value correction. *mulea* offers various methods, including Bonferroni and Benjamini–Hochberg *p*-value correction, and empirical false discovery rate (eFDR).

However, Bonferroni and Benjamini–Hochberg methods assume independent tests, which rarely holds true in functional enrichment analyses. For example, GO categories exhibit a hierarchical structure, potentially leading to unnecessary exclusion of significant results (enriched ontology entries). Therefore, *mulea* implements the robust, resampling-based eFDR, which takes into account the distribution of test statistics, making it better suited for analysing gene sets and ontologies typically employed by biologists. The eFDR implementation is based on the method described by Reiner et al*.* [14] and Hastie et al*.* [13].

##### The empirical false discovery rate (efdr)

For each ontology entry (*j* = *1,2,…,J*) and the investigated *target set* (*e.g.*, significantly differentially expressed genes), *mulea* calculates a *p*-value (*p*_*j*_) based on the hypergeometric test. To assess the unbiased statistical significance of each ontology entry, we compute the empirical false discovery rate (*eFDR*_*j*_) using a resampling-based approach.

First, we determine the *rank* of each ontology entry's *p*-value relative to the *p*-values of all ontology entries. *R*_*j*_ refers to the rank of the *p*-value of the *j*^*th*^ ontology entry. Here, we do note the *indicator function* with *Iverson brackets*: *I()*:

$${R}_{j}={\sum }_{i=1}^{J}I\left({p}_{i}\le {p}_{j}\right) , j=1...J$$

To calculate the expected rank ($${\overline{R}}_{j}$$) of the *p*-value of the *j*th ontology entry, we apply a resampling strategy, where resampling steps are indexed with *s* = *1,2,…,S*. In each resampling step, we generate a *simulated target set* with the same size as the *original target set*, but with randomly selected elements from the *background set*. Then we recalculate the hypergeometric tests and the ranks of the *p*-values ($${R}_{j}^{s})$$ for each resampling step. Let $${\overline{R}}_{j}$$ be the expectation of the $${R}_{j}^{s}$$ values over *s*:

$${\overline{R}}_{j}=\frac{{\sum }_{s=1}^{S}{R}_{j}^{s}}{S}$$

The eFDR of the *j*th ontology entry (*eFDR*_*j*_) is calculated as the ratio of the expected rank (${\overline{R}}_{j}$) to the actual rank (*R*_*j*_). If the calculated *eFDR*_*j*_ exceeds 1, it is truncated to 1.

$${eFDR}_{j}=min\left(\frac{{R}_{j}}{{\overline{R}}_{j}}, 1\right)$$

To enhance clarity, a simplified R code illustrating the functional enrichment test and eFDR calculations is provided in the Supplementary Note.

Calculating the eFDR for all ontology entries is computationally demanding, especially for large ontologies and numerous resampling steps (*mulea* recommends at least 10,000). To significantly improve the processing speed, *mulea* implements the eFDR functionality in efficient C +  + code which can be run on multiple threads. While similar approaches exist in tools like *Gowinda* [15] and *FuncAssociate* [16], *mulea* offers advantages in terms of data type compatibility and offline usability.

#### The ranked list-based gene set enrichment analysis (GSEA)

*Mulea* facilitates ranked list-based enrichment analysis using the GSEA approach. This method requires an ordered list of elements (*e.g.*, genes or proteins) as input, where the order reflects the user's prior analysis (*e.g.*, *p*-values and/or fold-changes). The list should encompass all elements involved in the analysis, such as all expressed genes in a differential expression study. *mulea* leverages the Kolmogorov–Smirnov statistic coupled with a permutation test [5] to assess enrichment within gene sets. This implementation is achieved through the integration of the *fgsea* Bioconductor package [17].

### Input types and formats

In the *mulea*, the overrepresentation analysis is implemented in the ora function which requires three inputs: (1) an ontology (*e.g.*, GO) data frame that fits the investigated taxa and the applied gene or protein identifier type; (2) a vector containing the list of elements under investigation (*e.g.*, differentially expressed genes), named as target set; and (3) a vector containing the background set of elements for the analysis (*e.g.*, all genes that were investigated in the experiment). Likewise, the gsea function, which calculates the enrichment of ranked elements, requires three inputs: (1) an ontology data frame; (2) a vector containing the names or identifiers of the elements (genes or proteins); and (3) a vector containing values corresponding to their order (*e.g., p*-values or log fold-change values).

#### Ontologies

*Mulea* R package offers a rich collection of ontologies to enhance functional enrichment analyses. These ontologies span across diverse species: *Arabidopsis thaliana*, *Bacillus subtilis*, *Bacteroides thetaiotaomicron*, *Bifidobacterium longum*, *Bos taurus*, *Caenorhabditis elegans*, *Chlamydomonas reinhardtii*, *Danio rerio*, *Daphnia pulex*, *Dictyostelium discoideum*, *Drosophila melanogaster*, *Drosophila simulans*, *Escherichia coli*, *Gallus gallus*, *Homo sapiens*, *Macaca mulatta*, *Mus musculus*, *Mycobacterium tuberculosis*, *Neurospora crassa*, *Pan troglodytes*, *Rattus norvegicus*, *Saccharomyces cerevisiae*, *Salmonella enterica*, *Schizosaccharomyces pombe*, *Tetrahymena thermophila*, *Xenopus tropicalis*, *Zea mays*, and cover various biological concepts:

1. Gene Ontology (GO): Ontologies are available for 13 species, ranging from *Escherichia coli* bacteria to humans, and are provided as separate ontologies for investigating biological processes, cellular components, and molecular functions. Additionally, combined versions for all three aspects are included.
2. Pathway databases: Pathway information from various resources like Pathway Commons [18], Reactome [19], SignaLink [20], and WikiPathways [21] is incorporated for 14 species, providing insights into molecular networks.
3. Transcription factor regulation: This category integrates data from ATRM [22], dorothEA [23], RegulonDB [24], TFLink [25], TRRUST [26], and Yeastract [27] databases, covering the regulatory influence of transcription factors for 8 species.
4. MicroRNA regulation: For 8 species, *mulea* offers microRNA regulation data sourced from miRTarBase [28], aiding in understanding microRNA-mediated gene regulation.
5. Gene expression data: Specifically for fruit flies (*Drosophila melanogaster*), gene expression data from Flyatlas [29] and Modencode [30] is included.
6. Genomic location data: This category, downloaded from Ensembl [31], provides detailed chromosomal location information (bands and consecutive genes) for 26 species.
7. Protein domain content: Protein domain information retrieved from the PFAM database [32] is available for 24 species, facilitating the identification of functional domains within sets of proteins.

The wide range of ontologies we provide, are stored in a standardised GMT format. These ontologies are also accessible through the *muleaData* ExperimentData Bioconductor package for added convenience. To cater to various research needs, the ontologies are available in all major gene and protein identifiers, including UniProt protein ID, Entrez ID, Gene Symbol, and Ensembl gene ID. We applied the API of UniProt (https://www.uniprot.org/help/id_mapping) to map between different identifiers. For direct access, 879 pre-defined GMT files are hosted on a dedicated GitHub repository (https://github.com/ELTEbioinformatics/GMT_files_for_mulea).

The GMT format contains collections of genes or proteins associated with specific ontology entries in a tab-delimited text file. The GMT file can be read to R as a data frame using the read_gmt function of *mulea* or the ExperimentHub identifier of the ontology using the *muleaData* package. Each term is represented by a single row in the GMT file and in the data frame as well. Each row includes three types of elements: (1) The ontology identifier, referred to as “ontology_id”, which uniquely identifies the element within the file or data frame. (2) The ontology name or description, referred to as “ontology_name”, provides a user-friendly label or textual description that clarifies the meaning of each term. (3) A list of associated gene or protein identifiers (separated by spaces) belonging to each term.

Additionally, in the GMT files, comment lines starting with "#" provide supplementary information about the referenced ontology: *e.g.*, type, source, species, version, and identifier type. Similar information is also available in the *muleaData* package when using the query function of the ExperimentHub library [33]. We recommend using the "primary identifiers" (listed in each GMT file and the Supplementary Table 1) for each ontology whenever possible. It is important to note that using alternative identifiers might lead to slightly different enrichment results due to potential inconsistencies in identifier coverage.

To enhance the biological interpretation of genomic and proteomic ndata, *mulea*'s adoption of the standardised GMT format allows the seamless integration of external data resources. This includes established databases like MsigDB [5] and Enrichr [6], as well as KEGG pathways (a conversion script is available in the https://github.com/ELTEbioinformatics/GMT_files_for_mulea GitHub repository). Notably, *mulea* accommodates user-defined ontologies, facilitates the translation between list variables and data frames with the list_to_gmt function, and has a dedicated function (write_gmt) to save GMT files, for further expanding its analytical capabilities.

##### Refining enrichment analysis by filtering ontology entries

Enrichment analysis results can sometimes be skewed by overly specific or too broad ontology entries. *mulea* empowers users to address this issue by enabling the exclusion of such entries from the analysis. This filtering capability allows researchers to ensure that the results better match the expected scope.

## Results

The mulea R package addresses the limitations of traditional enrichment analysis methods by offering a comprehensive and flexible toolkit. It tackles the issue of overcorrected *p*-values through a robust empirical false discovery rate (eFDR) method, specifically designed for interconnected biological data. *mulea* also goes beyond existing tools (Table 1) by incorporating a wide range of ontologies encompassing Gene Ontology, pathways, regulatory elements, genomic locations, and protein domains. This versatility allows researchers to tailor their enrichment analysis to specific questions, such as identifying enriched transcriptional regulators or overrepresented protein domains.

**Table 1:** Comparing the functionalities of *mulea* to other functional enrichment R packages and web servers

Name	Type	Nr. of species	Nr. of ontology types	Can supply custom ontology	Empirical false discovery rate
*mulea*	R package	27	22	✓	✓
clusterProfiler [8]	R package	Uses various databases		✓	✗
Enrichr [6, 34]	web server	2	227	✗	✗
fenr [35]	R package	As in the GO database	5	✗	✗
fgsea [17]	R package	Uses the msigdbr package [5]		✗	✗
gprofiler2 [7]	R package	Uses the g:profiler database		✗	✗
GOfuncR [36]	R package	As in the GO database	1	✗	✗
GOstats [37]	R package	35	2	✗	✗
Metascape [38]	web server	10	18	✗	✗
OpenXGR [39]	web server	1	24	✓	✗
topGO [40]	R package	Undetermined	1	✗	✗
WebGestalt and WebGestaltR [41]	R package & web server	12	6	✗	✗

### Demonstrating the differences between the results when applying the eFDR and different *p*-value correction methods

To elucidate the disparities between the eFDR and conventional *p*-value correction methods, we conducted an overrepresentation analysis using a test dataset of significantly upregulated *Escherichia coli genes* (GSE55662 Gene Expression Omnibus experiment [42]). The Regulon database [24], containing transcription factors and their target genes, provided the ontology for this analysis. We evaluated the overrepresentation of transcription factors targeting these upregulated genes, excluding those with fewer than 3 or more than 400 targets.

**Fig. 1:**
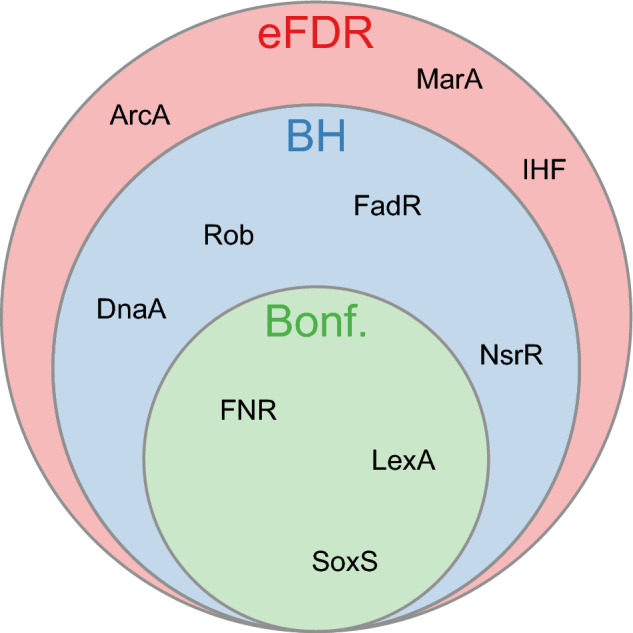
Overlap of transcription factors identified using the eFDR and different *p*-value correction methods. The Venn diagram shows the transcription factors whose target genes are overrepresented (eFDR of corrected *p*-value < 0.05) among significantly upregulated *Escherichia coli* genes (GSE55662 Gene Expression Omnibus experiment), using the Regulon database [24]. Transcription factors regulating less than 3 or more than 400 target genes were excluded. The diagram illustrates the overlap between transcription factors significantly overrepresented when using the eFDR (red), the Benjamini-Hochberg (abbreviated as BH, blue), and the Bonferroni (abbreviated as Bonf., green) *p*-value correction methods with the threshold < 0.05. The size of each section indicates the count of transcription factors specific to that method or shared between methods

Utilising the ora function from the *mulea* package, we independently applied the eFDR analysis, the Benjamini-Hochberg, and the Bonferroni corrections to the raw *p*-values. A comparison of the significant results (where the eFDR or corrected *p*-value < 0.05, Supplementary Table 2) revealed that conventional *p*-value corrections (Benjamini–Hochberg and Bonferroni) tend to be overly conservative, leading to a reduction in the number of significant transcription factors compared to the eFDR. As illustrated in Fig. 1, by applying the eFDR we were able to identify 10 transcription factors, while with the Benjamini–Hochberg and Bonferroni corrections only 7 and 3, respectively. This suggests that the eFDR may be a more suitable approach for controlling *false* positives in this context.

### The eFDR method outperforms Benjamini–Hochberg *p*-value correction for interconnected biological data

To evaluate the performance of the empirical false discovery rate (eFDR) *versus* the Benjamini–Hochberg *p*-value correction method in overrepresentation analysis, we conducted a simulation study. We generated *artificial target gene sets* using genes from randomly selected source ontology entries. Tests were marked as *true positives* if the source ontology entries were found among significantly overrepresented results. Other significantly enriched ontology entries were counted as *false positives*.

We first identified an ontology that shows especially high overlaps (number of common genes) between its entries, and therefore is most affected by the overcorrection of *p*-values when applying the Benjamini–Hochberg correction. Our analysis identified the transcription factor—target gene interactions dataset for budding yeast (*Saccharomyces cerevisiae*) from the Yeastract database [27] as having the highest overlap between ontology entry pairs (mean number of overlapping genes: 16.23, compared to the overall mean of all ontologies: 1.03).

**Fig. 2:**
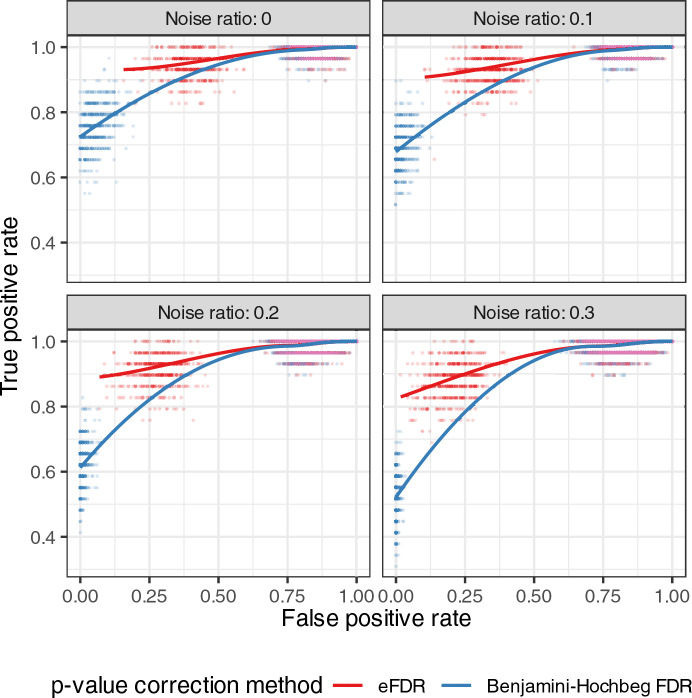
Performance comparison of eFDR and Benjamini–Hochberg *p*-value correction method, using on a highly overlapping ontology. The scatter plot displays the true positive rate (*y*-axis) *versus* the false positive rate (*x*-axis) for different noise ratios (0, 0.1, 0.2, and 0.3), using the Yeastract database budding yeast transcription factor—target gene interactions GMT file which has the highest mean overlap between ontology entry pairs. Each data point represents the true and false positive rates of a single simulation experiment. Lines show local polynomial regression fits for each method, with red and blue colours representing eFDR and Benjamini–Hochberg results, respectively

We randomly selected 20% of the ontology entries from this dataset as source entries. To simulate biological data with *noise*, we used 85% of the genes associated with the source entries as the *core target gene set* and added other genes at various ratios (0, 0.1, 0.2, and 0.3). In the overrepresentation analysis, all genes in the ontology were used as the background set. To calculate the eFDR we applied 1000 resampling steps in each round. Finally, we investigated if the enrichment analysis recovered the original randomly chosen source ontology entries and calculated the *true and false positive rates* for each result in the case of the Benjamini–Hochberg *p*-value correction and the eFDR (Fig. 2). Each noise ratio scenario was repeated 1000 times, resulting in a total of four million individual analyses (4 noise ratios × 1000 iterations with 1000 repeats each for eFDR calculation).

The overlapping entry calculator, simulation and plotting scripts are available on the https://github.com/ELTEbioinformatics/mulea_eFDR_testing GitHub repository.

Our simulations consistently revealed a significant improvement in true positive rates when employing the eFDR compared to the Benjamini–Hochberg method. This observation was statistically confirmed by one-sided paired *t*-tests, with all *p*-values falling below 2.2e−16 across the tested noise ratios. These results suggest that the eFDR method better captures the underlying structure of the data compared to the Benjamini–Hochberg approach.

To investigate how the performance of the empirical false discovery rate compares to the Benjamini–Hochberg *p*-value correction method under varying conditions, we repeated our previous analyses using an ontology with a low mean overlap (0.9) between ontology entry pairs. We employed a GMT file containing budding yeast transcription factor-target gene interactions, measured using small-scale methods, obtained from the TFLink database. Our findings revealed that while the eFDR still yielded a higher rate of true positives than the Benjamini–Hochberg correction, the performance differences between the two methods were less pronounced but remained statistically significant (Supplementary Figure).

### Visualisation of results

**Fig. 3:**
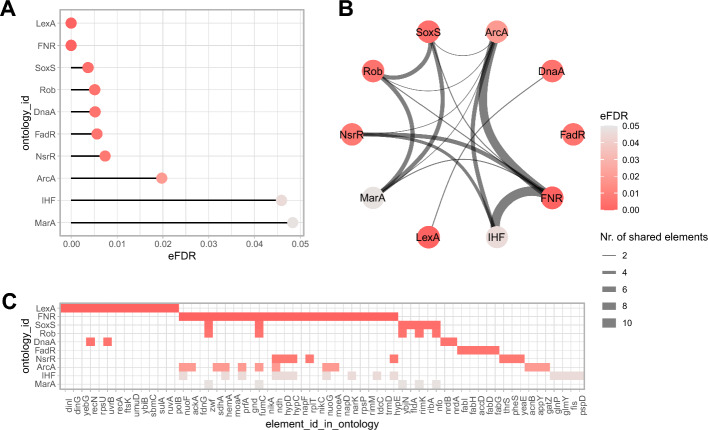
Visualization examples for an overrepresentation analysis. Visualisation of transcription factors whose target genes are overrepresented among significantly upregulated *Escherichia coli* genes (GSE55662 Gene Expression Omnibus experiment), using the Regulon database [24]. Transcription factors regulating less than 3 or more than 400 target genes were excluded. **A** Lollipop chart: visualises the distribution of eFDR values (*x*-axis) < 0.05 for enriched transcription factors (*y*-axis). **B** Network representation: nodes represent enriched transcription factors (coloured based on eFDR values), while edges connect nodes sharing at least one target gene among the significantly upregulated genes, and are weighted by the number of such shared genes. **C** Heatmap: illustrates which elements (target genes, *x*-axis) belong to enriched ontologies (transcription factors, *y*-axis). Cell colours correspond to eFDR values

*Mulea* offers various formats for presenting enrichment analysis results. By default, both ORA and GSEA results are provided in a tabular format. Additionally, users can leverage the *mulea* package to generate diverse visualisations, including lollipop and barplot charts, networks, and heatmaps (Fig. 3).

## Discussion and conclusions

Here we present the *mulea* R package, offering a unique combination of features for functional enrichment analysis. *mulea* integrates two enrichment approaches (ORA and GSEA) with an empirical false discovery rate (eFDR) correction method, providing robust statistical assessments. Additionally, *mulea* encompasses diverse ontologies for enrichment analysis across multiple species, data types, and identifiers, catering to a broad range of research needs. While some functionalities overlap with existing software (Table 1), *mulea* presents a comprehensive solution, uniting advanced methods and gene sets within a single package. This streamlined approach simplifies the analysis process and facilitates the interpretation of high-throughput results. While *mulea* shares functional similarities with tools like Enrichr, g:Profiler, and clusterProfiler, it offers several distinct advantages. Notably, compared to these tools, *mulea* implements a more appropriate multiple-testing correction method (eFDR) making the analysis more sensitive to detect significant enrichments in commonly used biological ontologies. Furthermore, *mulea* provides pre-defined gene sets for a broad range of organisms by including 27 species. Thus, *mulea* extends beyond an established gene set collection, MSigDB, which is limited to human and mouse. This broader species coverage enhances the applicability of *mulea* to various research contexts. Furthermore, *mulea* empowers users to incorporate their own ontologies using a dedicated function, enabling them to leverage datasets from diverse sources and extend the analytical scope beyond the default options. These unique features establish *mulea* as a versatile and user-friendly resource for researchers conducting functional enrichment analyses.

## Supplementary information

Supplementary material 1

Supplementary material 2

Supplementary material 3

Supplementary material 4

## Data Availability

The scripts and datasets generated and analysed during the current study are available in the following GitHub repositories: (1) the mulea R package: https://github.com/ELTEbioinformatics/mulea; (2) the muleaData ExperimentData Bioconductor package: https://github.com/ELTEbioinformatics/muleaData; (3) the GMT files and the scripts to create them: https://github.com/ELTEbioinformatics/GMT_files_for_mulea; and (4) the scripts for the simulation: https://github.com/ELTEbioinformatics/mulea_eFDR_testing.

## References

[1] Dennis G, Sherman BT, Hosack DA, Yang J, Gao W, Lane HC, et al. DAVID: Database for Annotation, Visualization, and Integrated Discovery. Genome Biol. 2003;4:P3.12734009

[2] Zhang B, Kirov S, Snoddy J. WebGestalt: an integrated system for exploring gene sets in various biological contexts. Nucleic Acids Res. 2005;33:W741-748. 15980575 10.1093/nar/gki475 PMC1160236

[3] The Gene Ontology Consortium. The Gene Ontology resource: enriching a GOld mine. Nucleic Acids Res. 2021;49:D325–34. 33290552 10.1093/nar/gkaa1113 PMC7779012

[4] Kanehisa M, Furumichi M, Sato Y, Ishiguro-Watanabe M, Tanabe M. KEGG: integrating viruses and cellular organisms. Nucleic Acids Res. 2021;49:D545–51. 33125081 10.1093/nar/gkaa970 PMC7779016

[5] Subramanian A, Tamayo P, Mootha VK, Mukherjee S, Ebert BL, Gillette MA, et al. Gene set enrichment analysis: a knowledge-based approach for interpreting genome-wide expression profiles. Proc Natl Acad Sci. 2005;102:15545–50. 16199517 10.1073/pnas.0506580102 PMC1239896

[6] Chen EY, Tan CM, Kou Y, Duan Q, Wang Z, Meirelles GV, et al. Enrichr: interactive and collaborative HTML5 gene list enrichment analysis tool. BMC Bioinform. 2013;14:128. 10.1186/1471-2105-14-128 PMC3637064 23586463

[7] Raudvere U, Kolberg L, Kuzmin I, Arak T, Adler P, Peterson H, et al. g:Profiler: a web server for functional enrichment analysis and conversions of gene lists (2019 update). Nucleic Acids Res. 2019;47:W191–8. 31066453 10.1093/nar/gkz369 PMC6602461

[8] Wu T, Hu E, Xu S, Chen M, Guo P, Dai Z, et al. clusterProfiler 4.0: a universal enrichment tool for interpreting omics data. Innovation. 2021;2:100141. 34557778 10.1016/j.xinn.2021.100141 PMC8454663

[9] Krämer A, Green J, Pollard J Jr, Tugendreich S. Causal analysis approaches in ingenuity pathway analysis. Bioinformatics. 2014;30:523–30. 24336805 10.1093/bioinformatics/btt703 PMC3928520

[10] Ari E, Ölbei M, Gul L, Bohár B. muleaData: genes sets for functional enrichment analysis with the “mulea” R package. 2024.

[11] Bonferroni CE. Teoria statistica delle classi e calcolo delle probabilità. 8th edition. Florence, Italy: Seeber; 1936.

[12] Benjamini Y, Hochberg Y. Controlling the false discovery rate: a practical and powerful approach to multiple testing. J R Stat Soc Ser B Methodol. 1995;57:289–300.

[13] Hastie T, Tibshirani R, Friedman J. The elements of statistical learning: data mining, inference, and prediction. 2nd ed. New York: Springer; 2009.

[14] Reiner A, Yekutieli D, Benjamini Y. Identifying differentially expressed genes using false discovery rate controlling procedures. Bioinformatics. 2003;19:368–75. 12584122 10.1093/bioinformatics/btf877

[15] Kofler R, Schlötterer C. Gowinda: unbiased analysis of gene set enrichment for genome-wide association studies. Bioinformatics. 2012;28:2084–5. 22635606 10.1093/bioinformatics/bts315 PMC3400962

[16] Berriz GF, Beaver JE, Cenik C, Tasan M, Roth FP. Next generation software for functional trend analysis. Bioinformatics. 2009;25:3043–4. 19717575 10.1093/bioinformatics/btp498 PMC2800365

[17] Korotkevich G, Sukhov V, Budin N, Shpak B, Artyomov MN, Sergushichev A. Fast gene set enrichment analysis. bioRxiv. 2021:060012.

[18] Rodchenkov I, Babur O, Luna A, Aksoy BA, Wong JV, Fong D, et al. Pathway Commons 2019 Update: integration, analysis and exploration of pathway data. Nucleic Acids Res. 2020;48:D489–97. 31647099 10.1093/nar/gkz946 PMC7145667

[19] Jassal B, Matthews L, Viteri G, Gong C, Lorente P, Fabregat A, et al. The reactome pathway knowledgebase. Nucleic Acids Res. 2020;48:D498-503. 31691815 10.1093/nar/gkz1031 PMC7145712

[20] Csabai L, Fazekas D, Kadlecsik T, Szalay-Bekő M, Bohár B, Madgwick M, et al. SignaLink3: a multi-layered resource to uncover tissue-specific signaling networks. Nucleic Acids Res. 2022;50:D701–9. 34634810 10.1093/nar/gkab909 PMC8728204

[21] Martens M, Ammar A, Riutta A, Waagmeester A, Slenter DN, Hanspers K, et al. WikiPathways: connecting communities. Nucleic Acids Res. 2021;49:D613–21. 33211851 10.1093/nar/gkaa1024 PMC7779061

[22] Jin J, He K, Tang X, Li Z, Lv L, Zhao Y, et al. An *Arabidopsis* transcriptional regulatory map reveals distinct functional and evolutionary features of novel transcription factors. Mol Biol Evol. 2015;32:1767–73. 25750178 10.1093/molbev/msv058 PMC4476157

[23] Garcia-Alonso L, Holland CH, Ibrahim MM, Turei D, Saez-Rodriguez J. Benchmark and integration of resources for the estimation of human transcription factor activities. Genome Res. 2019;29:1363–75. 31340985 10.1101/gr.240663.118 PMC6673718

[24] Tierrafría VH, Rioualen C, Salgado H, Lara P, Gama-Castro S, Lally P, et al. RegulonDB 11.0: comprehensive high-throughput datasets on transcriptional regulation in *Escherichia coli* K-12. Microb Genomics. 2022;8:000833. 10.1099/mgen.0.000833 PMC9465075 35584008

[25] Liska O, Bohár B, Hidas A, Korcsmáros T, Papp B, Fazekas D, et al. TFLink: an integrated gateway to access transcription factor–target gene interactions for multiple species. Database. 2022;2022:baac083. 36124642 10.1093/database/baac083 PMC9480832

[26] Han H, Cho J-W, Lee S, Yun A, Kim H, Bae D, et al. TRRUST v2: an expanded reference database of human and mouse transcriptional regulatory interactions. Nucleic Acids Res. 2018;46:D380–6. 29087512 10.1093/nar/gkx1013 PMC5753191

[27] Teixeira MC, Monteiro PT, Palma M, Costa C, Godinho CP, Pais P, et al. YEASTRACT: an upgraded database for the analysis of transcription regulatory networks in *Saccharomyces cerevisiae*. Nucleic Acids Res. 2018;46:D348–53. 29036684 10.1093/nar/gkx842 PMC5753369

[28] Huang H-Y, Lin Y-C-D, Cui S, Huang Y, Tang Y, Xu J, et al. miRTarBase update an informative resource for experimentally validated miRNA–target interactions. Nucleic Acids Res. 2022;2022(50):D222–30. 10.1093/nar/gkab1079 PMC8728135 34850920

[29] Chintapalli VR, Wang J, Dow JAT. Using FlyAtlas to identify better *Drosophila melanogaster* models of human disease. Nat Genet. 2007;39:715–20. 17534367 10.1038/ng2049

[30] The Modencode Consortium, Roy S, Ernst J, Kharchenko PV, Kheradpour P, Negre N, et al. Identification of functional elements and regulatory circuits by *Drosophila* modENCODE. Science. 2010;330:1787–97. 21177974 10.1126/science.1198374 PMC3192495

[31] Martin FJ, Amode MR, Aneja A, Austine-Orimoloye O, Azov AG, Barnes I, et al. Ensembl 2023. Nucleic Acids Res. 2023;51:D933–41. 36318249 10.1093/nar/gkac958 PMC9825606

[32] Mistry J, Chuguransky S, Williams L, Qureshi M, Salazar GA, Sonnhammer ELL, et al. Pfam: The protein families database in 2021. Nucleic Acids Res. 2021;49:D412–9. 33125078 10.1093/nar/gkaa913 PMC7779014

[33] Morgan M, Carlson M, Tenenbaum D, Arora S, Oberchain V, Morrell K, et al. ExperimentHub: client to access ExperimentHub resources. R package version 2.10.0. 2024.

[34] Evangelista JE, Xie Z, Marino GB, Nguyen N, Clarke DJB, Maayan A. Enrichr-KG: bridging enrichment analysis across multiple libraries. Nucleic Acids Res. 2023;51:W168–79. 37166973 10.1093/nar/gkad393 PMC10320098

[35] Gierlinski M. fenr: Fast functional enrichment for interactive applications. R package version 1.0.5. 2022.

[36] Grote S. GOfuncR: Gene Ontology enrichment using FUNC. R package version 1.22.2. 2024.

[37] Falcon S, Gentleman R. Using GOstats to test gene lists for GO term association. Bioinformatics. 2007;23:257–8. 17098774 10.1093/bioinformatics/btl567

[38] Zhou Y, Zhou B, Pache L, Chang M, Khodabakhshi AH, Tanaseichuk O, et al. Metascape provides a biologist-oriented resource for the analysis of systems-level datasets. Nat Commun. 2019;10:1523. 30944313 10.1038/s41467-019-09234-6 PMC6447622

[39] Bao C, Wang S, Jiang L, Fang Z, Zou K, Lin J, et al. OpenXGR: a web-server update for genomic summary data interpretation. Nucleic Acids Res. 2023;51:W387–96. 37158276 10.1093/nar/gkad357 PMC10320191

[40] Alexa A, Rahnenfuhrer J. topGO: enrichment analysis for Gene Ontology. R package version 2.54.0. 2023.

[41] Liao Y, Wang J, Jaehnig EJ, Shi Z, Zhang B. WebGestalt 2019: gene set analysis toolkit with revamped UIs and APIs. Nucleic Acids Res. 2019;47:W199-205. 31114916 10.1093/nar/gkz401 PMC6602449

[42] Méhi O, Bogos B, Csörgő B, Pál F, Nyerges Á, Papp B, et al. Perturbation of iron homeostasis promotes the evolution of antibiotic resistance. Mol Biol Evol. 2014;31:2793–804. 25063442 10.1093/molbev/msu223 PMC4166929

