# Supplementary material for: mulea: An R package for enrichment analysis using multiple ontologies and empirical false discovery rate
Source: BMC Bioinformatics. 2024 Oct 18;25:334. doi: 10.1186/s12859-024-05948-7 (PMC11490090; doi:10.1186/s12859-024-05948-7)
Supplement: Supplementary file 1 — Supplementary material 1 [file 12859_2024_5948_MOESM1_ESM.pdf]

Supplementary Note

for

mulea - an R package for enrichment analysis using multiple ontologies and empirical false discovery rate

Cezary Turek, Márton Ölbei, Tamás Stirling, Gergely Fekete, Ervin Tasnádi, Leila Gul, Balázs Bohár, Balázs Papp, Wiktor Jurkowski & Eszter Ari

Simplified R code of the functional enrichment test and eFDR calculations

Here `R_obs` refers to R_j and `R_exp` to $\overline{R}_j$.

```
#xxxxxxxxxxxxxx
# Function for functional enrichment test and eFDR calculation
#xxxxxxxxxxxxxx

# snow and rlecuyer packages are required

# Arguments of hypergeometric_test_with_eFDR function:
# number_of_permutations: the rounds of simulations (a single number),
#                         10,000 is recommended
# background_element_names: background genes (character vector)
# element_names: target genes to investigate (character vector)
# gmt: the ontology used for enrichment analysis (character list)
# nthreads: number of threads to use (a single number)

hypergeometric_test_with_eFDR <- function(number_of_permutations,
                                          background_element_names,
                                          element_names,
                                          gmt,
                                          nthreads = 4) {
  gmt_names <- names(gmt)
  num_gmt <- length(gmt)
  size_background_element_names <- length(background_element_names)
  size_element_names <- length(element_names)
  gmt_in_element_names <- integer(num_gmt)
  gmt_in_background_element_names <- integer(num_gmt)
  Genes_in_gmt <- integer(num_gmt)
  P_val <- double(num_gmt)
```

```r
R_obs <- integer(num_gmt)
# for every gmt entity in the gmt list
for (i in 1:num_gmt) {
  # create a vector of genes connected to the i-th gmt category
  gmt_i <- gmt[[i]]
  # hypergometric test data
  # q: number of common genes between element_names and a gmt entry
  gmt_in_element_names[i] <- length(intersect(element_names, gmt_i))
  # m: number of common genes between background_element_names and a gmt entry
  gmt_in_background_element_names[i] <- length(
    intersect(background_element_names, gmt_i))
  Genes_in_gmt[i] <- length(gmt_i)
  # hypergometric test
  P_val[i] <- 1 - phyper(
    q = gmt_in_element_names[i]-1,
    m = gmt_in_background_element_names[i],
    # n: number of non-background_element_names genes among a gmt entry
    n = size_background_element_names - gmt_in_background_element_names[i],
    # k: number of genes in element_names
    k = size_element_names)
}
# important for the precision of '0' is R
P_val_round <- round(P_val, digits = 15)
for (i in 1:num_gmt) {
  R_obs[i] <- sum(P_val_round <= P_val_round[i])
}
P_val_df <- data.frame(gmt_names,
                       gmt_in_element_names,
                       gmt_in_background_element_names,
                       Genes_in_gmt,
                       P = P_val,
                       R_obs)
#xxxxxx
# Simulation
#xxxxxx
R_exp <- integer(num_gmt)
# random sampling from background_element_names (background genes)
require(snow)
require(rlecuyer)
# max number of seeds for RNGstream is 6
seeds <- sample(seq(1e4, 1e6), 6)
# start the cluster
cl <- makeCluster(nthreads, type = "SOCK")
clusterSetupRNG(cl, type = 'RNGstream', seed = seeds)
P_Sim_vec <- clusterApply(cl,
                          rep(ceiling(number_of_permutations / nthreads),
```

```
                                    nthreads),
                                hypergeometric_test_simulation,
                                background_element_names,
                                element_names,
                                gmt,
                                P_val_df$gmt_in_background_element_names)
  # stop the cluster
  stopCluster(cl)
  P_Sim_vec <- as.vector(unlist(P_Sim_vec))
  P_Sim_round <- round(P_Sim_vec, digits = 15)
  for (l in 1:length(P_val_df$P)) {
    R_exp[l] <- sum(P_Sim_round <= P_val_round[l])
  }
  P_val_df$R_exp <- R_exp / number_of_permutations
  P_val_df$eFDR <- P_val_df$R_exp / R_obs
  return(P_val_df)
}

#xxxxxx
# Simulation function
#xxxxxx
hypergeometric_test_simulation <- function(number_of_permutations,
                                           background_element_names,
                                           element_names,
                                           gmt,
                                           gmt_in_background_element_names) {
  gmt_names <- names(gmt)
  num_gmt <- length(gmt_names)
  P_Sim_mat <- matrix(numeric(num_gmt * number_of_permutations),
                      ncol = number_of_permutations)
  size_background_element_names <- length(background_element_names)
  size_element_names <- length(element_names)
  for (j in 1:number_of_permutations) {
    Rand.element_names <- sample(background_element_names, size_element_names)
    for (i in 1:num_gmt) {
      gmt_i <- gmt[[i]]
      # hypergometric test
      P_Sim_mat[i, j] <- 1 - phyper(
        length(intersect(Rand.element_names, gmt_i)) - 1,
        gmt_in_background_element_names[i],
        size_background_element_names - gmt_in_background_element_names[i],
        size_element_names)
    }
  }
  return(as.vector(P_Sim_mat))
}
```

```r
#xxxxxxxxxxxxxx
# Application example
#xxxxxxxxxxxxxx

# need mulea to read and filter the gmt file
library(mulea)

# reading the GMT file from the GitHub repository
tf_ontology <-
read_gmt("https://raw.githubusercontent.com/ELTEbioinformatics/GMT_files_for_mulea/mai
n/GMT_files/Escherichia_coli_83333/Transcription_factor_RegulonDB_Escherichia_coli_Gen
eSymbol.gmt")
# filtering the ontology
tf_ontology_filtered <- filter_ontology(gmt = tf_ontology,
                                        min_nr_of_elements = 3,
                                        max_nr_of_elements = 400)
# taget set
target_set <-
readLines("https://raw.githubusercontent.com/ELTEbioinformatics/mulea/master/inst/extd
ata/target_set.txt")
# background set
background_set <-
readLines("https://raw.githubusercontent.com/ELTEbioinformatics/mulea/master/inst/extd
ata/background_set.txt")

# run the test
ora_results <- hypergeometric_test_with_eFDR(
  number_of_permutations = 10000,
  background_element_names = background_set,
  element_names = target_set,
  gmt = tf_ontology_filtered_list,
  nthreads = 8)
```
