# Supplementary material for: mulea: An R package for enrichment analysis using multiple ontologies and empirical false discovery rate
Source: BMC Bioinformatics. 2024 Oct 18;25:334. doi: 10.1186/s12859-024-05948-7 (PMC11490090; doi:10.1186/s12859-024-05948-7)
Supplement: Supplementary file 4 — Supplementary material 4 [file 12859_2024_5948_MOESM4_ESM.pdf]

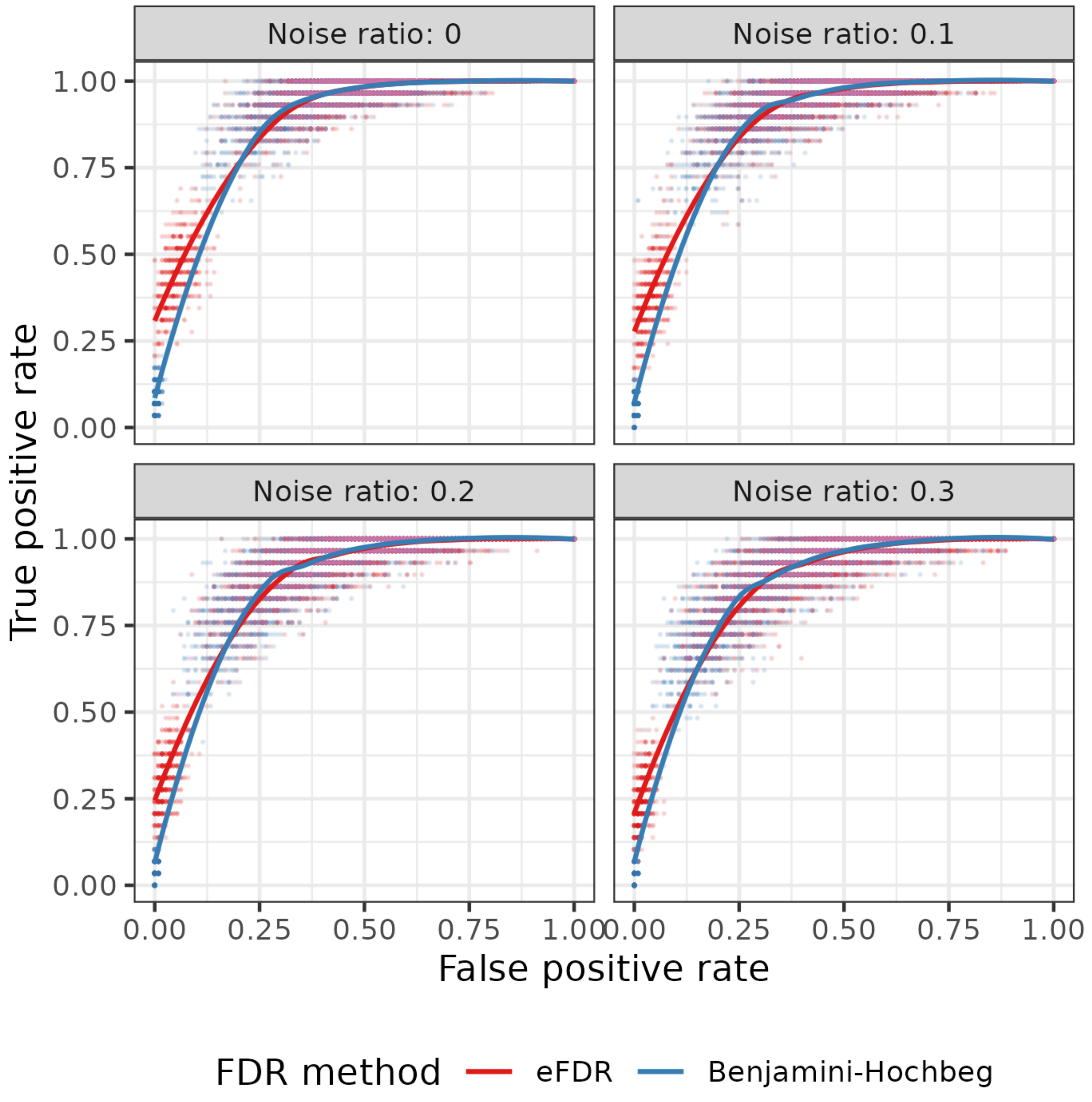

Supplementary Figure: Performance comparison of *eFDR* and Benjamini-Hochberg *p*-value correction method, using a lowly overlapping ontology. This scatter plot displays the true positive rate (*y*-axis) *versus* the false positive rate (*x*-axis) for different noise ratios (0, 0.1, 0.2, and 0.3). We applied the GMT file of budding yeast transcription factor - target gene interactions, measured with small-scale methods, downloaded from the TFLink database. This ontology has a low mean overlap (0.9) between ontology entry pairs. On the scatter plot, each data point represents the true and false positive rates of a single simulation experiment. Lines show local polynomial regression fits for each method, with red and blue colours representing eFDR and Benjamini-Hochberg results, respectively.
